# A study of bioactive glass–ceramic's mechanical properties, apatite formation, and medical applications

**DOI:** 10.1039/d2ra03235j

**Published:** 2022-08-16

**Authors:** Andualem Belachew Workie, Shao-Ju. Shih

**Affiliations:** Faculty of Materials Science and Engineering, Bahir Dar Institute of Technology, Bahir Dar University P. O. Box 26 Bahir Dar Ethiopia; Department of Materials Science and Engineering, National Taiwan University of Science and Technology 43 Sec. 4 Keelung Road Taipei 10607 Taiwan; Department of Fragrance and Cosmetic Science, Kaohsiung Medical University No. 100, Shih-Chuan 1^st^ Road Kaohsiung 80708 Taiwan d10904804@mail.ntust.edu.tw shao-ju.shih@mail.ntust.edu.tw

## Abstract

Apparently, bioactive glass–ceramics are made by doing a number of steps, such as creating a microstructure from dispersed crystals within the residual glass, which provides high bending strength, and apatite crystallizes on surfaces of glass–ceramics when calcium ions are present in the blood. Apatite crystals grow on the glass and ceramic surfaces due to the hydrated silica. These materials are biocompatible with living bone in a matter of weeks, don't weaken mechanically or histologically, and exhibit good osteointegration as well as mechanical properties that are therapeutically relevant, such as fracture toughness and flexural strength. As part of this study, we examined mechanical properties, process mechanisms involved in apatite formation, and potential applications for bioactive glass–ceramic in orthopedic surgery, including load-bearing devices.

## Abstract 2

Bioactive glass–ceramics are made by several steps, such as creating a microstructure from dispersed crystals within the residual glass, which provides high bending strength, and apatite crystallizes on surfaces of glass–ceramics with calcium ions.

## Graphical abstract

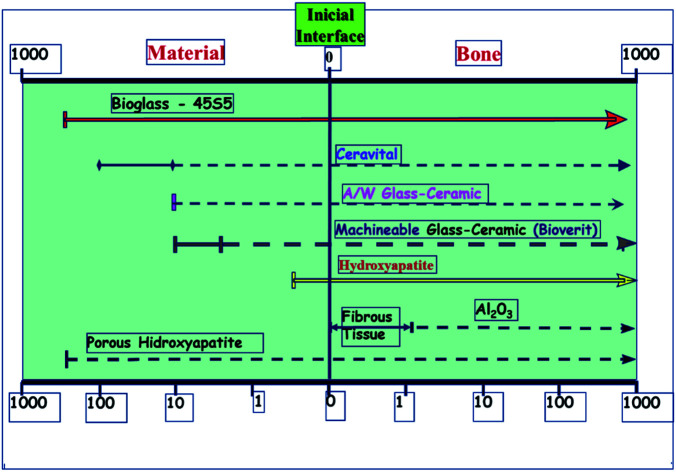

## Introduction

Ceramics have made remarkable progress in the last few decades for improving the quality of life of people. This revolution has produced ceramics manufactured and designed to repair and replace biological parts that are damaged, aged, or underperforming. Ceramics used for this purpose are known as bio ceramics.^1^ Ceramics are also utilized to replace circulatory system components, particularly heart valves. Unique glass compositions are also used therapeutically in the treatment of malignancies. Hip and knee replacements, heart valves, and dental root implants^2^ are just a few of the implants that are now commonplace and well-known to the general population.^3,4^ Bio ceramics are created in a variety of stages. Nearly inert ceramics (sapphire or zirconia), porous ceramics (hydroxyapatite), glass (Bioglass®), glass ceramics (A/W glass–ceramic), or composites (polyethylene-hydroxyapatite) are commonly used for these applications.^5^ Apatite is called after the Greek word apát, which means deception since apatite is frequently misidentified as a variety of other minerals. Because of its flexible framework structure, the apatite lattice can easily withstand a wide range of ionic substitutions. Apatites are naturally present in rocks on Earth, and fluor- and hydroxyapatite (HAp) variations have recently been identified on the Moon's surface. Apatite is also the most important inorganic mineral. Because it is a component present naturally in vertebrate hard tissues, it has both biological and therapeutic implications. Ca_5_(PO_4_)_3_(OH) is the chemical formula for natural apatite's, with some “CO_3_^2−^ replacing PO_4_^3−^, F^−^ substituting OH^−^, and Na^+^ or Mg^2+^ replacing Ca^2+^ ions. Artificial HAp has been employed in a number of medical applications, including bone replacement, dental cement, and dental porcelains”.^6^ Nonetheless, sintered porous or even dense HAp bone implants frequently fail because to mechanical properties inferior to those of real bone.

Glass–ceramic systems are not one-component systems,^7^ and the crystal composition differs from the parent glass. As a result, the leftover glass in the glass–ceramic must be designed differently than the parent glass.^8^ S. Donald Stookey in the United States developed glass–ceramics^9^ by accident in 1953 as a result of a heat treatment oven failure. An 18th-century Frenchman, René-Antoine Ferchault de Réaumur, used crystallization to make porcelain in the 18th century. However, this feature was usually looked at as a flaw until a few magnificent art pieces were incorporated into its design.^10^ A high amount of materials has been produced by controlling nucleation and crystal growth processes for a multitude of industrial applications^11^ Glass–ceramics have been used for a variety of things, including cooking utensils, windows, fireplace doors, and kitchenware since they were discovered.^12^ In addition to dental implants and telescope mirrors, radomes for missiles, waste management matrices, and optical purposes, ceramics also have many important applications.

**Fig. 1:**
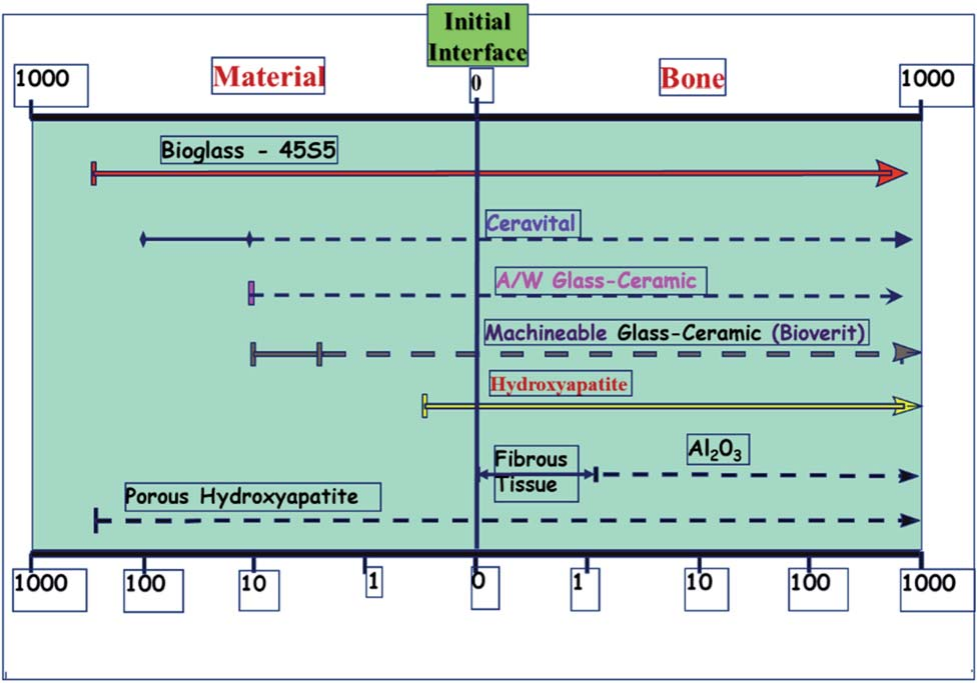
Commercial bio-ceramics interfacial thickness (mm).^5,18^

A glass–crystal composite may be obtained by the heating glass. The contents and sizes of the crystalline phase may be controlled. In comparison to parent glass and sintered ceramic,^13^ a glass–ceramic can outperform it; the mechanical strength of monophasic bioactive ceramics is higher.^14^ For example, the strength of bioglass-type glasses and sintered HAp is often less than that of human cortical bone. Kokubo *et al.* developed a glass crystallization method for making a similar composite in 1982.^15^ Reinforcing the endeavor was β-wollastonite (CaOSiO_2_), which has a silicate chain structure. Commercial bio-ceramics^16,17^ interfacial thickness (mm) is different depending on their mechanical properties and is justified as labeled in Fig. 1 below.

Bioactive ceramics that attach to bone must be chemically bonded to the bonelike apatite layer; this may change their bioactivity from A/W glass–ceramic to sintered HA due to the faster time for this layer to form.^19^ A/W ceramics are thought to release a significant amount of calcium and silicate ions into simulated body fluids, resulting in apatite formation on the surface of these objects. glasses and glass–ceramics provide calcium to the body, increasing apatite activity and silica on the glass surface, providing the conditions for nucleation, which results in the formation of apatite on these surfaces.^20,21^

The purpose of this study is to examine the mechanical properties of bioactive glass–ceramic materials and their formation of apatite, as well as how they can be incorporated into surgical instruments for treating bones in orthopedic surgery, such as loading bearing devices.

## Glass–ceramic microstructure formation

**Fig. 2:**
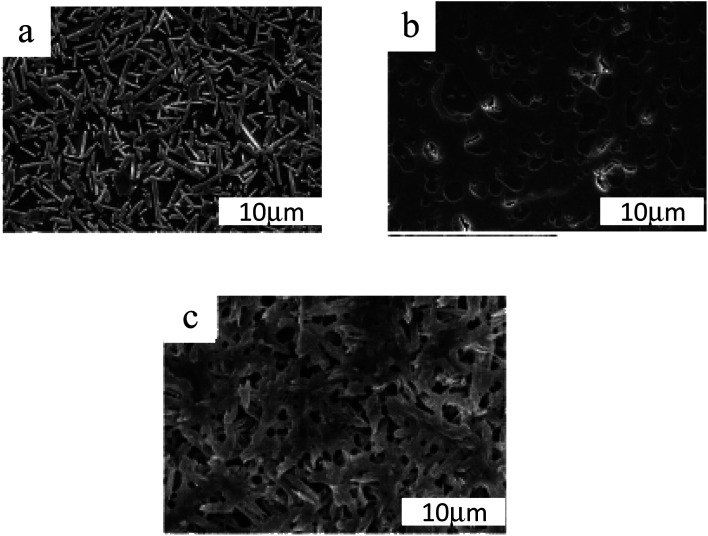
Typical SEM images of glass–ceramic microstructures treated by (a) 1400, (b) 800, and (c) 900 °C for 3 h.^22,23^

Glass–ceramics have exceptional characteristics due to their microstructure composed of homogeneously distributed crystals in a residual glass phase. Microstructures can have a range of features, as seen in Fig. 2. The percentage of crystals in any particular material can range from 20 to 90 vol%, with typical crystal sizes ranging from a few nm to a few microns.

Take notice of the various scales used in the micrographs.^24^ (a) Glass–ceramic Macro®. Mica is the primary crystalline phase. (b) Keralite® is a glass–ceramic material. (c) Kerawhite® is a glass–ceramic material. A chemical etching has exhibited the microstructure in these three glass–ceramic products made using volume nucleation.^25^ When heated, glass ceramics have a higher viscosity than glass precursors. As a result, two factors limit the change in viscosity^26^ with temperature: “the presence of crystals as well as the penetration of glass modifiers into the crystals during heating, leaving left over glass that is more viscous than its precursor”. In most cases, liquors that have a modest crystal concentration (a few percent by volume) are not referred to as glass ceramics, probably because crystallization does not have a significant impact on their viscosity. Photochromic glass lenses can be made with these glasses, which contain tiny crystals of silver halide that are exposed to light.^27^

A homogeneous crystallization of glasses^28^ is often impossible because the surface is lacking in nucleation or a defect, generating surface nucleation or large-scale nucleation.^29^ Using the surface nucleation technique, glass crystals are formed from a frit bound with binders and then heated under ceramic temperatures. They are then removed during the early thermal treatment phases.^30^ Sintering of the grains and then crystallization^31^ occur at higher temperatures. Crystallization is sometimes split into two stages. Having the ability to create early seeds and then to move on to the next stage that allows for the formation of the main crystal phase silicate glasses' nucleation phase is “typically 50 °C to 100 °C above the glass transition temperature”.^32^ In contrast, the development phase occupies a temperature range of 100 to 200 °C above the freezing point.

**Fig. 3:**
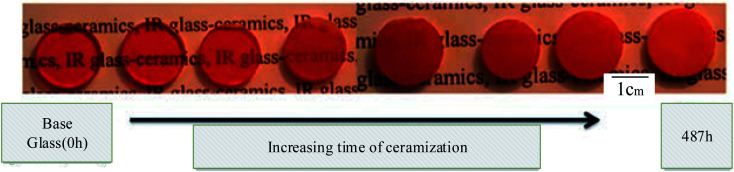
Typical systems and a description of how the glass–ceramic is produced as a function of thermal treatment time. This figure has been reproduced from ref. 33, with permission from Elsevier, Copyright ©2022.

Glass–ceramics may be produced in various glasses, not just silicate glasses, as long as this nucleation is accomplished, as shown above in Fig. 3 illustrated. The quantities of nucleating chemicals required vary significantly from system to system. They are generally 2 to 8 mol percent for oxides and fluorine and less than 1% for colloids.^34^

## Mechanical properties of glass–ceramics

**Fig. 4:**
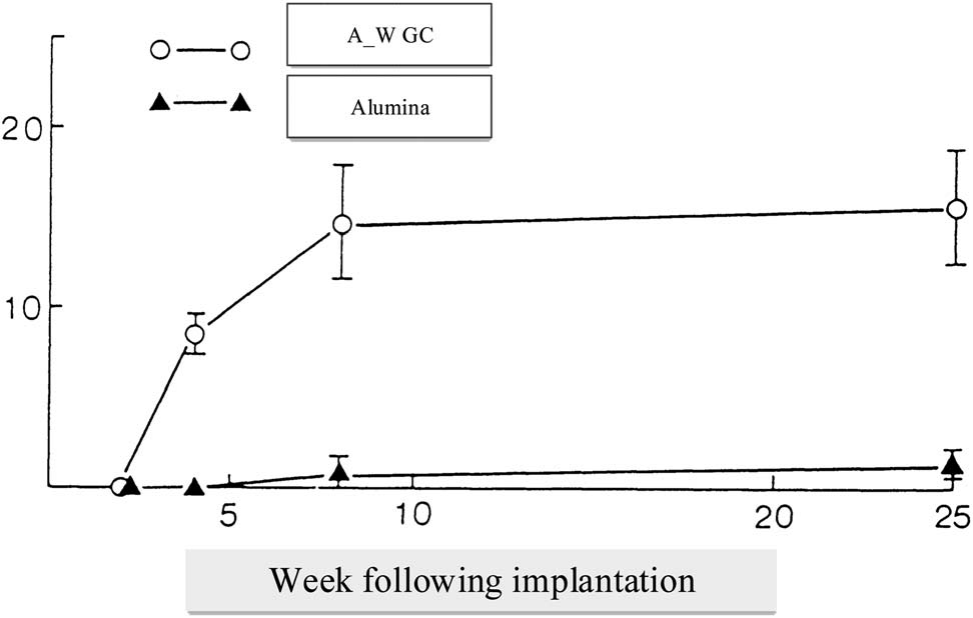
Tensile strength in kilograms per square centimeter (vertical axis) over implant time in weeks (horizontal axis).^5,41^

It is easy to shape A/W glass–ceramic with diamond cutters using screws; this glass–ceramic is approximately twice as strong as dense sintered HA (115 MPa) and even more substantial than a human cortical bone (160 MPa) in an airy environment (215 MPa).^35^ Bending strengths^36^ of the parent glass G and glass–ceramic A, which precipitate apatite solely, are 72 and 88 MPa, respectively. This suggests that A/W glass–ceramic's extraordinary bending strength is related to their high fracture toughness due to the precipitation of wollastonite and apatite.^37,38^ Glass–ceramic implants placed in the AW create a strong bond with the live bone and don't degrade in the body, mechanically and histopathologically.^39^ Having a glassy phase in the A/W glass–ceramic enables it to bond to bone more quickly than synthetic HA, possibly due to the release of more calcium ions after implantation, as shown in Fig. 4 which may trigger the formation of crystallized apatite nuclei.^40^

Thin sintered HA, glass–ceramic A, and glass–ceramic G should sustain continuous bending stress of 65 MPa for more than ten years, whereas A–W glass–ceramic should only last a minute. Some bio ceramics' failure loads^42^ after implantation is different, as shown below in Table 1 and bio ceramics with higher bioactivity,^43,44^ such as bioglass and cervical, are weaker than bone.

**Table 1:** Some bio ceramics' post-implant failure loads^3,44–61^

Materials	Failure load (kg)	Fracture location	Ref.
Alumina with high (*ρ*)	0.13 ± 0.02	Interface	45–48
Bioglass 45S5	2.75 ± 1.80	Within material	49–51
Ceravital (GC)	3.52 ± 1.48	Within material	52–54
Cerabone A/W (GC)	7.43 ± 1.19	Within bone	3 and 55–58
Hydroxyapatite	6.28 ± 1.58	Within material	59–62

Surface modifications such as Zr^+^ ion implantation can further minimize the degree of A/W glass–ceramic fatigue. This glass–ceramic, like other ceramics, displays a decrease in mechanical strength when loaded in an aqueous body environment due to stress corrosion-induced progressive fracture development. As shown below in Table 2 different bioceramics have varying properties depending on their surface energy and other mechanical properties.^63,64^

**Table 2:** Bioglass–ceramics' typical characteristics^64–85^

Bio ceramics	Flexural strength (MPa)	KIC MPa m^1/2^	*E* (GPA)	Bioactivity IB = 100/*t*50	Load to failure (kg)	Fracture location	Machinability	Ref.
Bio silicate	210	1.0	60	12	7.0–7.4*	Bone	Fair	65–68
45S5	70	0.6	50	12	2.8	Material	Poor	69–72
Cerabone (A/W)	215	2.0	220	3	7.4	Bone	Low	73–75
Ceravital	150	?	150	6	3.5	Material	Low	76–78
Hydroxyapatite	40–70	<1	120	2.5	6.2	Material	Low	79–82
Bioverit	160	1–2	90	3	??	??	Good	83–86

**Fig. 5:**
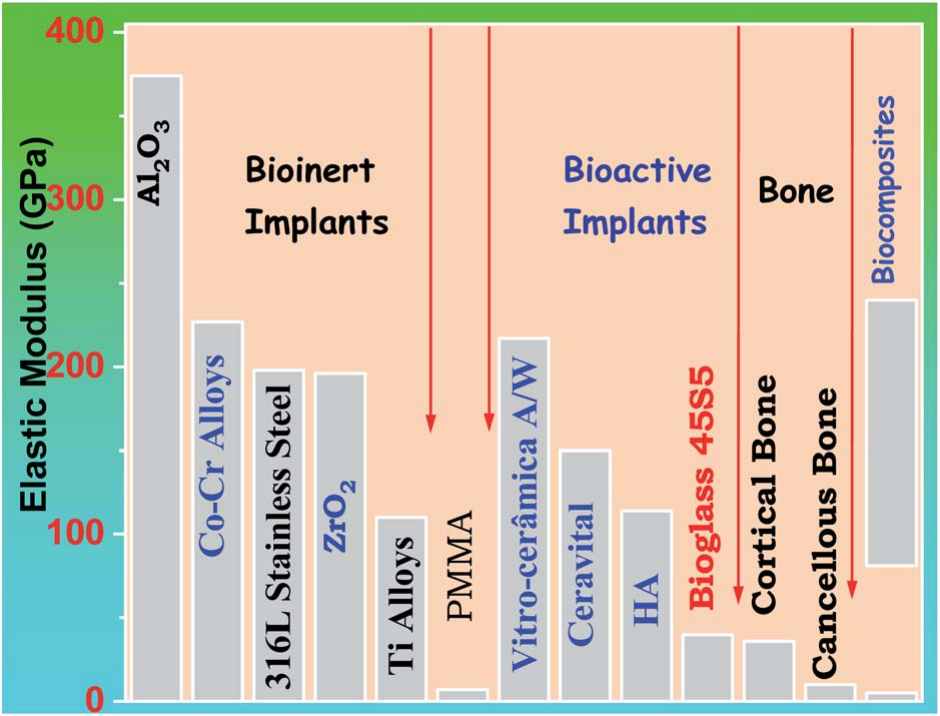
The elasticity modulus of prosthetic materials in comparison to bone^5,18^

Problems with 45S5 bioglass mechanical properties, Peitl and Zanotto altered the composition of 45S5, and it was feasible to create a glass–ceramic with identical biological behavior but far better mechanical properties.^87^ Bio silicate is currently ineligible for usage in load-bearing implants. It lacks sufficient mechanical properties. As shown in Fig. 5 below different prosthetic materials can have a comparison with bone in respect to the elastic modulus.

Glass G and glass–ceramic A^88^ have fracture surface energies of 3.3 and 6.4 Jm^2^, respectively. For A/W glass–ceramic, this high fracture energy helps explain its extraordinary fracture toughness.^89^ A/W glass–ceramic^90^ has a “roughened fracture surface, while glass G and glass–ceramic A have relatively smooth fracture surfaces”.^91,92^ Incredibly, wollastonite has such a powerful reinforcing effect while not being a fibrous substance. This suggests that wollastonite effectively prevents fractures from spreading straight rather than forcing them to turn or branch out. Table 3 shows the general properties of AW-GC and illustrates as follows.^93^ A/W glass ceramic^94,95^ decreases with decreasing stress rate in the presence of simulated body fluid at pH 7.25 at 36.5 °C that has ion concentrations comparable to that of human blood plasma, which is significantly lower than that of glass G.^96^ In A/W glass–ceramic, slow cracks are seen as 33. In glass–ceramic G, it has an average value of 9, while glass–ceramic A has an average value of 18 ^55,97^.

**Table 3:** Physical properties of AW_GC^35,93,97–112^

Physical properties	Corresponding measured values	Ref.
Density (g cm^−3^)	3.07	36 and 98–100
Strength in bending (MPa)	215	101–104
Strength in compression (MPa)	1080	105 and 106
Young's modulus (GPa)	118	107–109
Vickers hardness (HV)	680	110 and 111
Toughness to fracture (MPa^1/2^)	2.0	94
Slow crack expansion (*n*)	33	112 and 113

## Clinical application of glass–ceramic

**Fig. 6:**
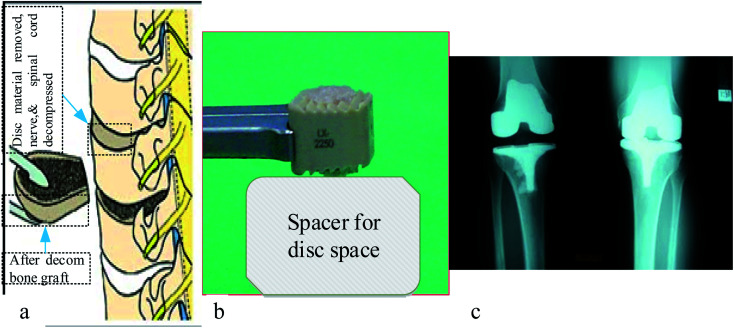
Anterior cervical discectomy illustration of (a) vertebral bodies (b) photo of bone graft substitute, and (c) X-ray result of implanted part.^119–121^

There are currently several ceramics available for the treatment of severe bone and joint illnesses or anomalies. Bio ceramics are used to replace significant volumes of bone loss due to medical diseases such as cancer.^114^ These may be rings concentric around a metallic pin put up the center of the residual bone itself.^115,116^ Because the pores in these implants are porous, new bone will grow into them, essentially functioning as a scaffold for new bone production, as seen in Fig. 6 shown below. The A–W glass–ceramic exhibits excellent osseointegration as well as therapeutically relevant mechanical properties,^117^ like fracture toughness and flexural strength. Furthermore, the system's inability to bulk nucleate and lack of bioresorbability provide further research and design problems. Although freshly generated chlorapatite glass ceramics exhibit the required resorbability and Osseo integration,^118^ more research is needed on their *in vivo* activity and structure–property connection, including microstructure and mechanical characteristics.

**Fig. 7:**
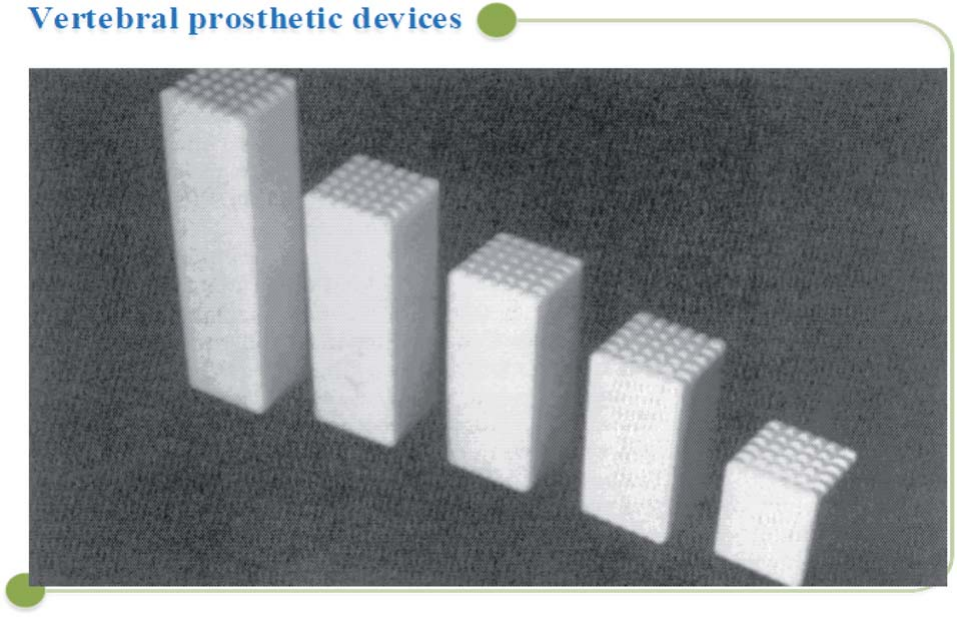
Vertebral prosthetic device selection.^5,41^

A/W glass–ceramic was aimed at using spine and hip surgeries of patients with severe lesions or bone abnormalities since 1983, but the mechanical strength of A/W glass–ceramic is not as good as that of cortical bone. There are no comparisons between hydroxyapatite and A/W glass–ceramics regarding mechanical strength,^117^ with a compressive strength of 10 800 kg cm^−2^ and a bending strength of 2000 kg cm^−2^ powerful. A/W glass ceramic^122,123^ has chemical compositions of Mg O 4.6, Ca O 44.9, SiO_2_ 34.2, and CaF_2_ 0.5 in weight percent. Calcium oxyfluorapatite (CaO_10_(PO_4_)_6_(O, F_2_)) and calcium silicate (CaSiO_3_) comprise 35, 40, and 25% of the total weight of each component, respectively. Several glass–ceramic vertebral prostheses have been developed for clinical use by Kokubo *et al.* (1986) to provide a stable, radiopaque anchor that bonds well to the bone. The prosthesis is available in various sizes, allowing the surgeon to choose the best one in the operating room, as seen in Fig. 7 below.

**Fig. 8:**
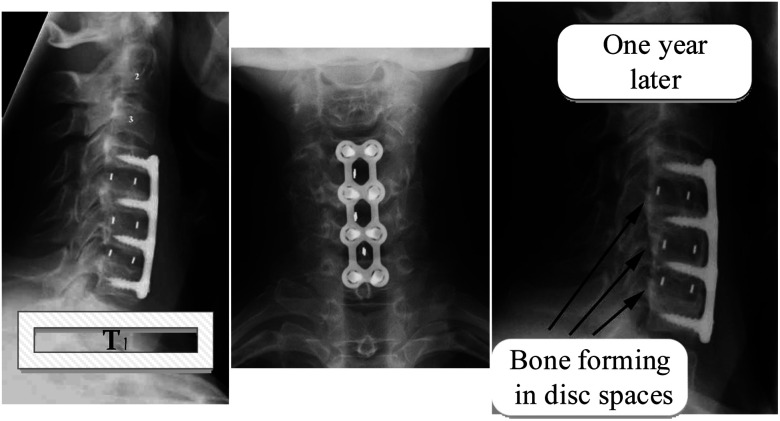
Bone development and bone-bonding.^119,129^

Alumina heads have been used for hip prosthetic crowns since 1965 because it is relatively strong and fracture-resistant, withstands severe mechanical loads, and is completely biocompatible. In orthopedics, zirconia-toughened alumina composites, particularly for knee replacements and hip prostheses, are increasingly used. Stronger than 4 MPa m^1/2^ and more rigid than 500–1500 GPa, these materials are offered in a range of strengths from 500–1500 GPa.^124^ A variety of applications for biomedical glass–ceramics is presently being explored because of their enhanced mechanical resistance or bioactivity, such as fillers in composites, dense pieces, or granules. A/W GC^25,125^ can be used to make thick blocks. This powder is fully densified at 830 °C before a heat treatment at 880 °C begins the crystallization of oxy-fluoro apatite (Ca_10_(PO_4_)_6_(O, F)_2_. A glass–ceramic material with a fracture toughness of approximately 2 MPa m^1/2^ and a fracture strength of about 210 MPa has fascinating mechanical properties.^126^ Interestingly, Cerabone® A/W features similar characteristics to glass–ceramics based on lithium disilicate, albeit it is considerably more bioactive.^127,128^ A glass–ceramic that is in contact with biological fluids releases Ca^2+^ ions, saturating the ceramic with bioactive properties, such as hydroxyapatite, calcium phosphates, and other forms of bioactive glass. Fig. 8 shows what happens when these materials are put in bone tissue. They stimulate bone formation and bind to the bone in varying degrees, showing how this is done.

This glass-surface ceramic also has a high concentration of silanol groups and apatite nucleation sites.^14^ Glass–ceramic surfaces develop a hydroxyapatite layer when these two properties are present. This layer can expand further due to the biological fluids' supply of Ca and P.^130,131^ A/W GC bonded to bone at a tightness equivalent to dense synthetic HAp, and its load was 70% that of bone. The A/W GC was attached to the bone with the same tightness as thick synthetic HAp, and its load was 70% that of the skeleton. A/W GC groups had a primarily fractured bond in the bone, compared with the HAp groups, which had a fractured principally bond in the ceramic; the bonded interface in neither group was disrupted, though.^132^

**Fig. 9:**
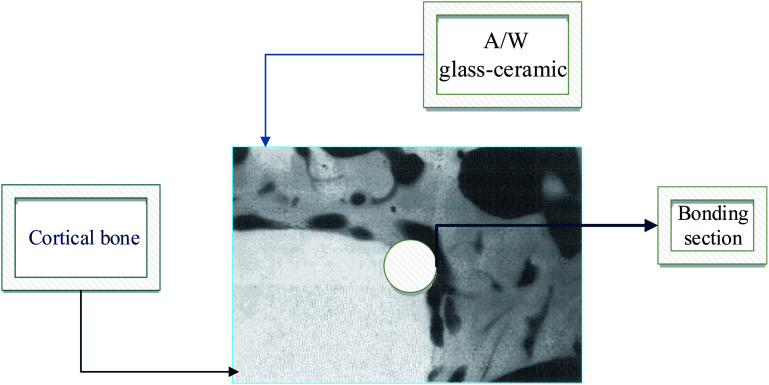
A/W Glass–ceramic linked to the bone.^5,41^

In Fig. 9 we show how A/W glass–ceramic, like synthetic HAp, soon binds to bone tissue when it is in contact with it by creating a Ca/P rich layer.

## Apatite layer formation on glass–ceramics

**Fig. 10:**
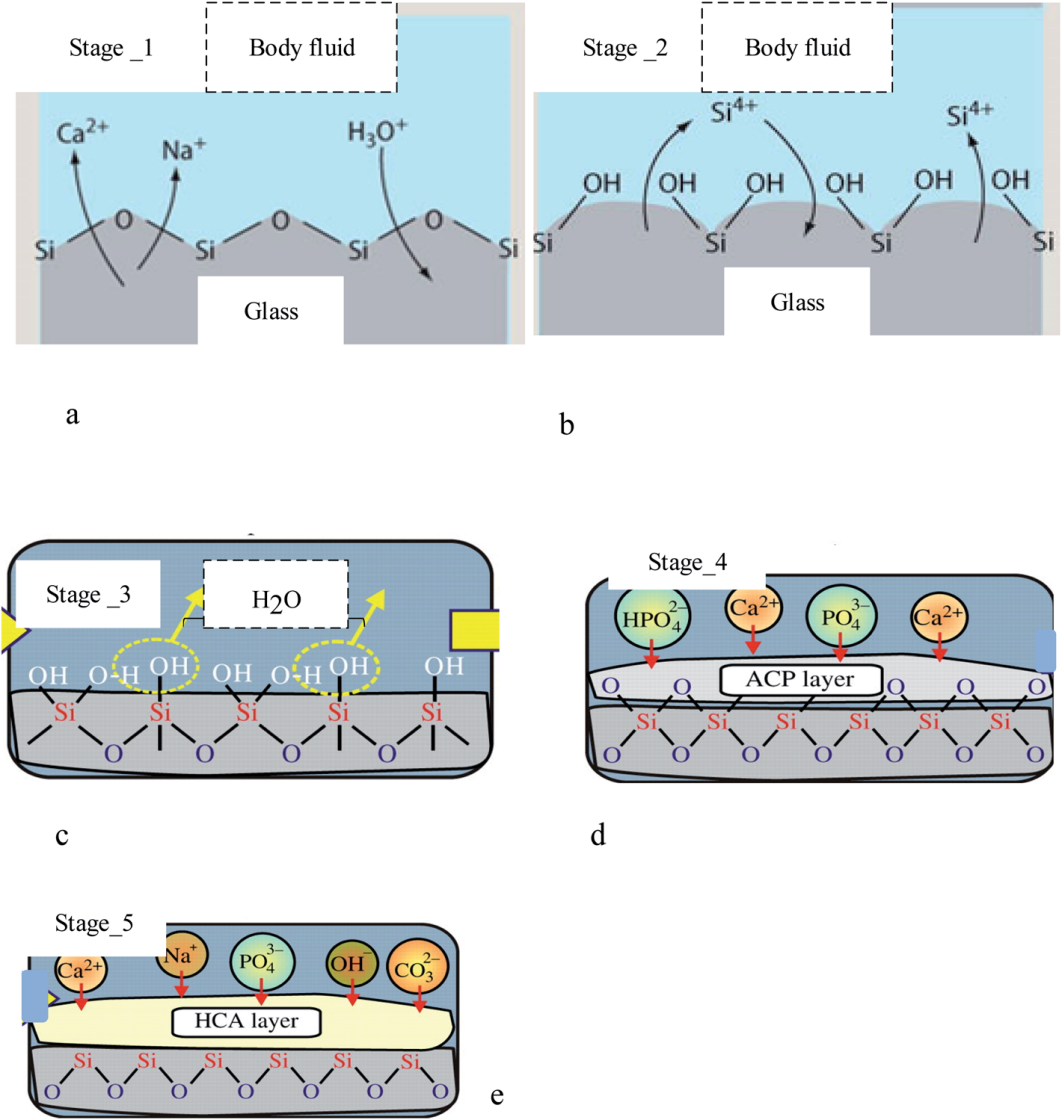
Formation stages of apatite on diverse substrates, including: (a) stage 1: fast ion exchange Na^+^ and Ca^2+^ ions with H^+^ from solution, (b) stage 2: breaking Si–O–Si bonds Si(OH)_4_ to solution, (c) stage 3: re-polymerization of a SiO_2_-rich layer, (d) stage 4: migration Ca^2+^ and PO_4_^3−^ to surface SiO_2_-rich layer forming amorphous CaO–P_2_O_5_ rich (ACP) film, and (e) crystallization ACP film by incorporation OH^−^ and CO_3_^2−^ from solution forming hydroxycarbonate–apatite.^18,58,138^

An *in vivo* generalized mechanism of apatite formation on surfaces of glass–ceramics and CaO–SiO_2_-based glasses has been suggested.^133^ When calcium ions are dissolved in the Glass and glass–ceramic surfaces, they increase the apatite ion activity in the surrounding body fluids.^134^ In addition, the hydrated silica on the surfaces of glasses and glass–ceramics allows for the nucleation of apatite.^56,135^ Although, as shown in Fig. 10, there was no silica gel layer present on the surface of the A/W glass–ceramic, “apatite nuclei” formed independently after absorbing calcium and phosphate ions^136^ from the surrounding body fluid.^137^ A significant fraction of silicate ions in the glass–ceramic dissolves into the simulated body fluid, which indicates that many silanol groups are present at the glass–ceramic surface.

Stage _2 of bioactive glasses compositions occurs even at neutral pH. From stage-3 H_2_O to solution, they were followed by Si-0-Si net regeneration in silica gel form. Type II glasses are ordered to test the following surface chemical reactions: as Fig. 10 stage I and stage II (to pH > 9). The bioactive glasses proceed through stage-1 and two and then engage in other surface chemical processes.^139^ Glass–ceramic implants constructed by five steps form a thin layer rich in calcium and phosphorus, which binds to surrounding bone and is found to be an apatite layer by micro X-ray diffraction.^52,140^

## Conclusion

Taking everything into account, this review covers the topics of bioactive glass–ceramics, microstructure development, mechanical characteristics, a method for apatite layer production on glass–ceramic surfaces, and medical applications. Glass–ceramic systems are not one-component systems, and the crystal composition differs from the parent glass. As a result, the leftover glass in the glass–ceramic must be designed differently than the parent glass. A glass–crystal composite may be obtained by heating glass. The content and size of the crystalline phase may be controlled, and have distinct challenges in surface coating since their thermal expansion coefficient does not match that of the substrate. In comparison to parent glass and sintered ceramic, a glass–ceramic can outperform it; the mechanical strength of monophasic bioactive ceramics is higher. When heated, glass–ceramics have a higher viscosity than glass precursors. As a result, two factors limit the change in viscosity with temperature: the presence of crystals as well as the penetration of glass modifiers into the crystals during heating, leaving behind glass that is more viscous than its precursor. Glass–ceramics have exceptional characteristics due to their microstructure composed of homogeneously distributed crystals in a residual glass phase. Microstructures can have a range of features. Glass homogeneous crystallization is frequently impossible because the surface lacks nucleation or has a defect, resulting in surface nucleation or large-scale nucleation. Using the surface nucleation technique, glass crystals are formed from a frit bound with binders and then heated under ceramic temperatures. This work review's glass–ceramic (apatite–wollastonite) exhibits excellent osseointegration and therapeutically acceptable mechanical properties, such as fracture toughness and flexural strength. Hence, bioactive glass–ceramics mostly possess higher mechanical properties than ordinary bioglass because of the presence of crystal phases formed, and hence they can be employed for load-bearing applications in medical applications for many years, and even substitute the use of metals in such applications, since their strength after a surface modification becomes enhanced, and that is why they are hot research issues in many research institutes. The author intends to show that the issue of biomaterials research, basically bioactive glass–ceramics research, continues to amaze and convey new concepts on the structure of solids covered, with an undeniable and potentially immense future. More study is needed to examine *in vivo* activity and the structure–property link, including the microstructure and mechanical properties.

## Author contributions

ABW: conceptualization, methodology, writing original draft, review & editing the whole paper. SJS: structure the overall framework of the paper, methodology, review, and edit the whole paper. All authors have read and agreed to the published version of the manuscript.

## Conflicts of interest

There are no conflicts to declare.

## Supplementary Material
